# What we can learn about biology from monogenic diseases; KIF11 and the glymphatic and lymphatic-like system of the retina and choroid and its possible role in the pathogenesis of pachychoroid and central serous chorioretinopathy

**DOI:** 10.1186/s40942-026-00950-y

**Published:** 2026-10-27

**Authors:** Jose S. Pulido, J. Fernando Arevalo, Lily C. Farrell, Justin C. Muste

**Affiliations:** 1 Mid Atlantic Retina, Wills Eye Hospital, Philadelphia, PA USA; 2 Wilmer Eye Institute, Johns Hopkins Medical Center, Baltimore, MD USA; 3 Department of Ophthalmology, University Hospital Waterford, Waterford, Ireland

## Abstract

**Background:**

A recent article by Farrell et al. describes changes in the retinal vasculature and choroidal vasculature in patients with pathogenic variants in KIF11. The concept of a glymphatic system in the retina and a lymphatic-like system in the choroid is still in discussion.

**Methods:**

Discussion of how this KIF11 variant helps to understand the glymphatic system in the retina and the lymphatic-like system in the choroid with review of the literature about the lymphatic-like system of the choroid and the glymphatic system of the retina.

**Results:**

Iowa single cell Spectacle shows that the VEGFR3/Flt4 is expressed in the choroid and that correlates with a few prior studies. Additionally, KIF11 is important in the expression of VEGFR3/Flt4.

**Discussion:**

The recent study showed how KIF11 affects the retinal vasculature and the choroidal vasculature. As KIF11 is important in the formation of the lymphatics, it probably is related in part to the development of a lymphatic-like system in the choroid and potentially the glymphatic system of the retina.

In a recent publication, Farrell et al. describe the choroidal and retinal findings in a small case series of patients with KIF11 pathogenic variants that cause an autosomal dominant genetic disorder characterized systemically by microcephaly and lymphedema [1]. In the eye, it can be associated with chorioretinal dysplasia, usually in the inferior retina, just below the optic disc (MCMLR-microcephaly, chorioretinal dysplasia, lymphedema and mental retardation OMIM #152950). In addition, there can be retinovascular changes similar to familial exudative vitreoretinopathy (FEVR) and recently Farrell et al. have shown that there is foveal hypoplasia as well. MCMLR is extremely rare but can it teach us about the biology of the retina, the choroid and the retinal vasculature? Rare monogenic diseases have provided insights into biological pathways that extend far beyond the diseases themselves. KIF11-associated retinopathy may represent another such example.

First, indulge us with a digression into other situations where a monogenic disease taught us about a retinal disease. In 1949, Linus Pauling and his colleagues showed that sickle cell disease was caused by a hemoglobinopathy and called it a “molecular disease” [2]. Subsequently, it was found that it was associated to one amino acid change in the beta globin in position 6 from glutamate which is polar to valine which is nonpolar. This change was sufficient to allow the beta globin to polymerize in hypoxic or acidic conditions. The polymerized hemoglobin then causes the red cells to sickle and the rheologic change then causes sludging [3, 4]. In the retinal vasculature as well as in other small vessels, the sludging red cells then can occlude the small vessels. Understanding the molecular basis of the clinical findings, allowed methods to treat the disease. Now there are CRISPR based methods to treat the hemoglobinopathy [5].

The same concept can be applied to an understanding of KIF11 chorioretinopathy. The Human Protein Atlas indicates that KIF11 is not commonly expressed in the eye but it is highly expressed in the bone marrow and lymphoid tissues (*Tissue expression of KIF11 - Summary - The Human Protein Atlas*) (Fig. 1). This was confirmed in another mRNA atlas (*Gene Expression - CZ CELLxGENE Discover*). However, given the established role of KIF11 in mitosis, its enrichment in these tissues may reflect their high proliferative activity rather than lymphatic identity per se. KIF11 may also exert important effects during early ocular development through pathways that remain incompletely understood. Importantly, these datasets do not include in utero expression but even in a fetal expression atlas, it is still expressed at a low level in microglia and Muller Cells (The Brotman Baty Institute (BBI) Human Gene Expression Atlas).

**Fig. 1:**
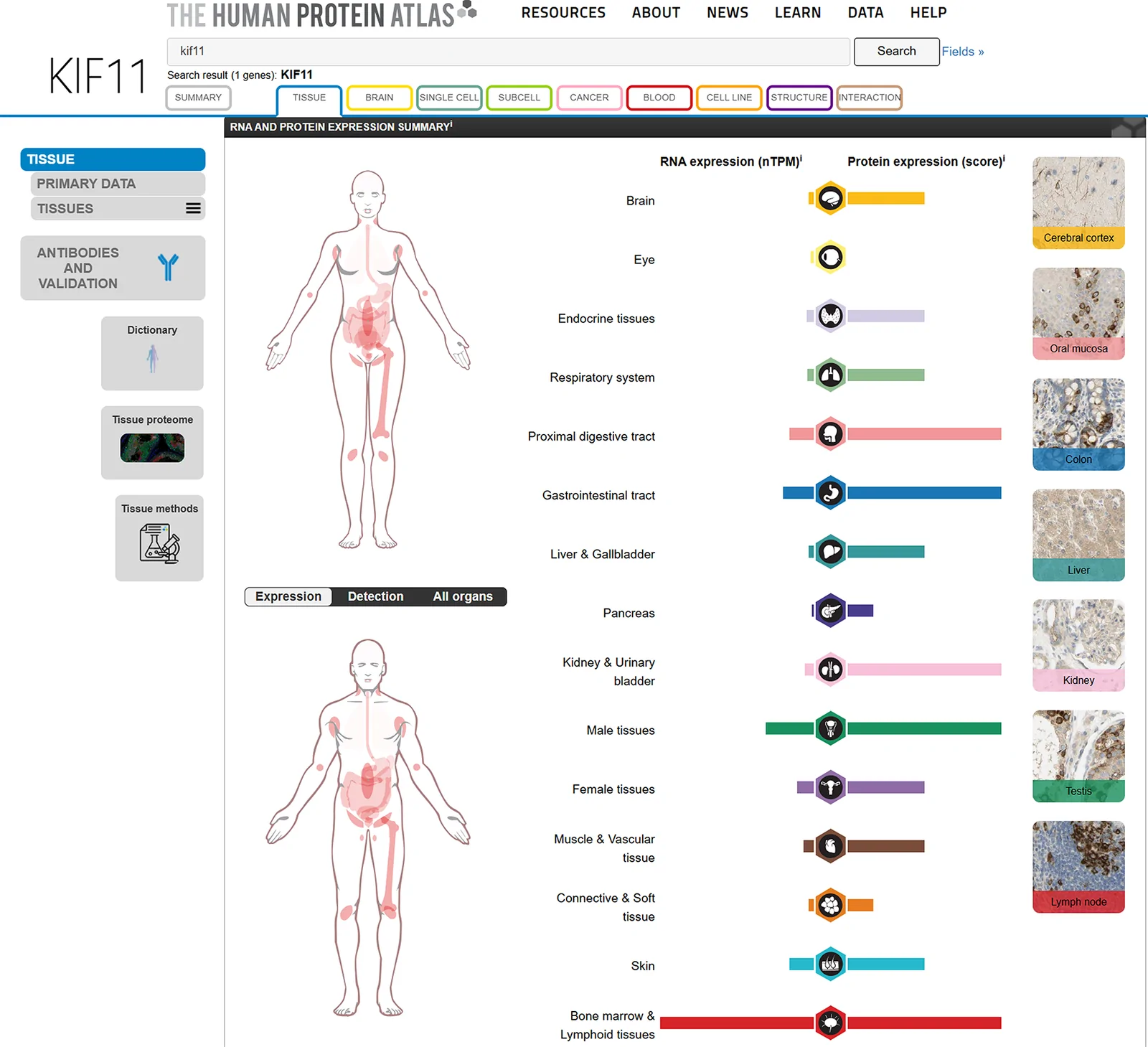
Human protein atlas RNA and protein expression in various tissues and organs. Note how it is highly expressed in the bone marrow and lymphoid tissues

**Fig. 2:**
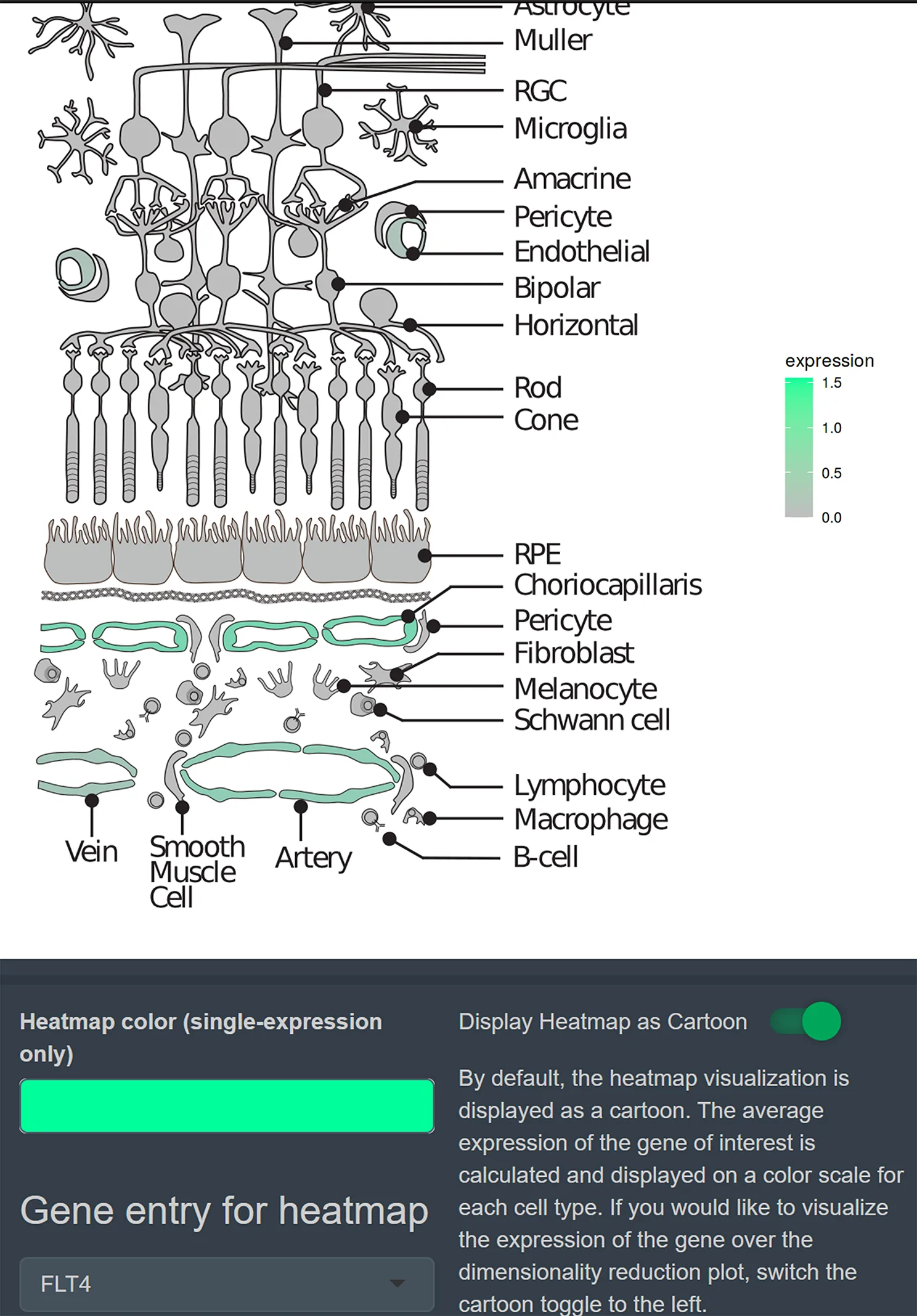
Green coloring demonstrates VEGFR3/FLT4 moderate mRNA expression in the choroid and mild expression in the retinal vasculature

How, therefore, might KIF11 contribute to choroidal development? Why does KIF11 cause chorioretinal dysplasia? KIF11 expresses a protein called EG5. EG5 is important in early development and growth of humans. It is a molecular motor, and, in the lymphatics, it is associated with VEGFR3 (FLT4). VEGFR3 is important in the evolution and expansion of the lymphatic system. Although the exact biochemical mechanism remains to be fully elucidated, the regulatory link between KIF11 and lymphatic development is functionally supported by data showing that KIF11 deficiency impairs the VEGFR3 signaling pathway [6]. This is further supported by Fig. 2, which demonstrates moderate VEGFR3/FLT4 mRNA expression in the choroid and mild expression in the retinal vasculature. (Fig. 2) https://singlecell-eye.org/app/spectacle/.

Neither the retinal vasculature nor the choroid have been thought to have a classic lymphatic system. Recently however, it has been determined that the central nervous system has a form of lymphatic system called the glymphatic system [7]. There is flow from the blood in the retina and a similar system occurs to drain metabolites and water and this has led to several articles that hypothesize the presence of the glymphatic system in the retina and a lymphatic-like system in the choroid with little actual evidence [8–12]. However, in a mouse model, Sauntharrajan et al. have demonstrated a lymphatic like drainage system in the choroid and the staining of this system using antibodies that stain VEGFR3/FLT4 [13]. Nevertheless, VEGFR3/FLT4 expression and lymphatic-associated features do not in themselves establish the presence of conventional, anatomically distinct lymphatic vessels within the human choroid. Accordingly, we use the term “lymphatic-like” to describe these molecular and functional similarities while acknowledging that the existence and anatomical organization of classical lymphatic vessels in the human choroid remain under investigation.

Considering that KIF11 is important in the development of the lymphatic system elsewhere, the fact that it is so closely associated with the functioning of VEGFR3 then understanding how a mutation in KIF11 causes microcephaly, lymphedema and chorioretinal dysplasia ultimately produces the choroidal malformation and the retinal vascular maldevelopment helps us understand the role of the glymphatic system in the retina and choroid.

In the article by Farrell et al., they show that the retinal vasculature has findings like those in FEVR with maldevelopment of the foveal avascular zone [1]. The choroid has evidence of areas where the choroid is thicker and also areas where the choroid is completely underdeveloped. The areas involved can be broad, but most tend to involve an area below the disc at 6 o clock which is the last area of the retina and choroid to close embryologically. It might be that more severe mutations cause wider maldevelopments than milder ones. Regardless, this KIF11 is important for the maturation of the choroid. These findings raise the possibility that altered KIF11–VEGFR3 signaling may contribute to retinal and choroidal developmental abnormalities.

It further leads to questions as to how a lymphatic-like system of the choroid might be affected by pharmacologic agents. High doses of corticosteroids or Cushing syndrome can cause sodium and water retention by increasing downstream aldosterone production. If the choroidal lymphatic-like system is maldeveloped and subsequently overloaded then from the Starling equation, fluid then extravasates into the interstitial spaces causing pachychoroid and/or central serous chorioretinopathy (CSCR) [14].With more minor elevations of corticosteroids for instance following intraarticular or inhaled corticosteroid injections, the pathophysiology is not as obvious and further studies on the role of the lymphatic-like system and CSCR are required. This proposed lymphatic-like mechanism should be considered complementary to, rather than a replacement for, established mechanisms implicated in CSCR and the pachychoroid spectrum, including mineralocorticoid receptor signaling and choroidal venous dysfunction. Altered lymphatic-like drainage, if present, may represent an additional contributor to impaired choroidal fluid homeostasis within this broader pathophysiological framework. The possibilities described in this paragraph are presently speculative considerations that deserve further evaluations to determine their possible veracity.

In conclusion, history has shown that rare monogenic disorders illuminate fundamental biological processes. KIF11-associated retinopathy may similarly provide important insight into retinal vascular development, choroidal maturation, and potentially the biology of glymphatic and lymphatic -like pathways within the eye.

## Data Availability

No datasets were generated or analysed during the current study.
